# Virtual Clinical Trials in 2D and 3D X-ray Breast Imaging and Dosimetry: Comparison of CPU-Based and GPU-Based Monte Carlo Codes

**DOI:** 10.3390/cancers14041027

**Published:** 2022-02-17

**Authors:** Giovanni Mettivier, Antonio Sarno, Youfang Lai, Bruno Golosio, Viviana Fanti, Maria Elena Italiano, Xun Jia, Paolo Russo

**Affiliations:** 1 Dipartimento di Fisica “Ettore Pancini”, Università di Napoli Federico II, I-80126 Napoli, Italy; 2 INFN Sezione di Napoli, I-80126 Napoli, Italy; sarno@na.infn.it (A.S.); mariaelenaita@hotmail.it (M.E.I.); paolo.russo@na.infn.it (P.R.); 3 Department of Radiation Oncology, University of Texas Southwestern Medical Center, Dallas, TX 75287, USA; youfang.lai@mavs.uta.edu (Y.L.); xun.jia@utsouthwestern.edu (X.J.); 4 Dipartimento di Fisica, Università di Cagliari, I-09042 Monserrato, Italy; golosio@unica.it (B.G.); viviana.fanti@ca.infn.it (V.F.); 5 INFN Sezione di Cagliari, I-09042 Monserrato, Italy

**Keywords:** virtual clinical trials, Geant4, GPU code, breast cancer

## Abstract

**Simple Summary:**

Virtual clinical trials in X-ray breast imaging may permit substantial reduction of the costs, times, and exposure risk to patient of clinical trials. Monte Carlo simulation techniques are increasingly adopted for VCT in breast imaging and dosimetry studies. This work aims to compare three different platforms for breast VCT studies, to develop real-time virtual DM, DBT and BCT examinations, where the in-silico image acquisition process takes a computational time comparable to that typical of a corresponding real clinical examination.

**Abstract:**

Computational reproductions of medical imaging tests, a form of virtual clinical trials (VCTs), are increasingly being used, particularly in breast imaging research. The accuracy of the computational platform that is used for the imaging and dosimetry simulation processes is a fundamental requirement. Moreover, for practical usage, the imaging simulation computation time should be compatible with the clinical workflow. We compared three different platforms for in-silico X-ray 3D breast imaging: the Agata (University & INFN Napoli) that was based on the Geant4 toolkit and running on a CPU-based server architecture; the XRMC Monte Carlo (University of Cagliari) that was based on the use of variance reduction techniques, running on a CPU hardware; and the Monte Carlo code gCTD (University of Texas Southwestern Medical Center) running on a single GPU platform with CUDA environment. The tests simulated the irradiation of cylindrical objects as well as anthropomorphic breast phantoms and produced 2D and 3D images and 3D maps of absorbed dose. All the codes showed compatible results in terms of simulated dose maps and imaging values within a maximum discrepancy of 3%. The GPU-based code produced a reduction of the computation time up to factor 10^4^, and so permits real-time VCT studies for X-ray breast imaging.

## 1. Introduction

In X-ray breast imaging, virtual clinical trials (VCTs)—i.e., computational reproductions of clinical exams—may provide a substantial reduction of the costs, investigation times, and exposure risk to the patient with respect to real clinical trials on a patient population [1]. VCTs may constitute a valid support in technological evaluations, for defining quality assurance protocols, and for testing and development of imaging units in breast cancer screening and diagnosis. VCTs implement in-silico replications of medical tests, including replicated apparatus and imaging generation chain as well as a digital model of the patient anatomy (e.g., voxelized phantoms). They should provide a fair digital reproduction of the image acquisition hardware, of the imaging setup, and produce realistic images for diagnosis. For this goal, it is fundamental to devise acceptable models of the replicated patient anatomy (the breast) via anthropomorphic digital phantoms [2] reproducing realistic anatomical noise and features in the simulated images.

Analytical approaches that are based on photon tracking ray-tracing algorithms are usually adopted in these simulations. They are very efficient computationally, but they usually do not consider scattered photons, whose effect is often considered ex post as an image pattern that is superimposed on the primary transmitted 2D image [3,4,5,6], for example analytical models for the simulation of single scatter events in cone beam CT have been developed (e.g., ref. [7]). On the other hand, Monte Carlo (MC) simulation techniques intrinsically model both the primary and scattered photon beams. They are increasingly adopted for breast imaging and dosimetry studies as they represent the solution of choice for imaging simulations in breast VCTs [1]. An example is the VICTRE project (Virtual Imaging Clinical Trials for Regulatory Evaluation) [7], a large VCT study comparing the performance of digital breast tomosynthesis (DBT) with respect to digital mammography (DM), for breasts with different anatomical characteristics.

The medical physics group of the University of Naples Federico II is carrying out a VCT project for 2D (DM) and 3D (DBT and breast computed tomography, BCT) imaging and dosimetry in breast cancer diagnostics using an MC code (Agata) that is based on the Geant4 toolkit [1]. In addition to imaging, the software calculates the 3D glandular dose maps and provide mean glandular dose (MGD) estimates [8]. One of the limitations of this Geant4-based code running on CPU (central processing unit)-based platforms is the long calculation time that is related to the large number of photon histories to be launched for low uncertainty level. Inefficiency of CPU-based MC codes also arises from handling photons one by one. Speeding up MC simulations is a goal of interest both for research purposes in imaging and for dosimetry in the clinical setting (e.g., for MC-based radiotherapy planning). The shorter the time linked to a single study, the greater the number of studies that can be carried out. To this end, several groups have developed GPU (graphics processing unit)-based MC codes, particularly for imaging applications [9,10,11,12,13,14,15], where the GPU hardware permits a very significant acceleration of the computation process and a drastic reduction of the hardware costs. This result is obtained thanks to the large number of cores of a single GPU card (for example the NVIDIA GeForce RTX^TM^ 3090 that was used in this study has 10,496 cores) with a price of a few thousand euros with respect to a typical CPU cluster (in this work we used a cluster with 36 CPU cores with a cost of tens of thousands of euros). 

This work aimed to compare three different platforms for in-silico X-ray imaging in breast VCT studies, evaluating their accuracy and timing performance, which are a critical feature of all MC simulation techniques. The three MC codes that were evaluated were: (1) the Geant4 code that was developed by the team at University & INFN Napoli (U Naples) within the Agata project, based on the Geant4 toolkit and running on a CPU hardware; (2) the XRMC MC code that was developed by University of Cagliari (U Cagliari) based on the use of variance reduction techniques, running on a CPU hardware; and (3) the MC code gCTD that was developed by University of Texas Southwestern (U Texas), running on a GPU platform and written in CUDA software language. Typical case studies for the evaluation of accuracy in terms of dosimetry and imaging will be simulated using the three platforms. The MC Agata code was used as benchmark for the evaluation. With reference to this investigative step, our final goal was to develop real-time virtual DM, DBT, and BCT examinations on anthropomorphic digital breast phantoms, devising in-silico image acquisition processes requiring a computational time that is comparable to that which is typical of a corresponding real clinical examination.

## 2. Materials and Methods

### 2.1. Monte Carlo Platforms

#### 2.1.1. Geant4—Agata (U Naples)

Agata is a CPU-based MC software platform for the simulation of DM, DBT, and BCT examinations. The software is based on the Geant4 simulation toolkit with the low energy physics list Option4 [1,8,16,17,18] and is an upgraded version of a previous code that was adopted for the calculation of MGD estimates and 3D glandular dose maps [1,8,19,20,21,22]. It simulates the photoelectric, coherent (Rayleigh), and incoherent (Compton) scatter photon interactions; electrons are not tracked but are supposed to release the dose at the point where the photoelectric and incoherent interactions occur. To compute the dose distribution, for each photon impinging on the scoring area, the code scores its energy, its 2D location, and the angle between the photon direction and the direction normal to the detector surface. To score the dose map within the organ, the energy deposition is scored together with the position of the interacting photon. Since the glandular tissue is considered the most radiosensitive tissue within the breast, only the dose that is deposited within the glandular tissue is considered. For this reason, the dose deposition is tallied only within the voxels that are classified as glandular tissue.

#### 2.1.2. XRMC (U Cagliari) 

The XRMC is a CPU-based program for the X-ray photon transport simulation which makes extensive use of variance reduction techniques [23]. It has been used with conventional X-ray sources [24,25,26,27,28] as well as inverse Compton scattering sources [29,30] for planning experimental setups and/or optimizing experimental parameters. The code was written in the C++ programming language and a description of the main variance reduction techniques that were used can be found in refs. [31,32,33,34]. The simulated interaction processes are the photoelectric effect (eventually followed by fluorescent emission) and the coherent and incoherent scattering. At each step of the simulation, the state of a photon is defined by its position vector, its direction its polarization, and its energy. The energy of the electrons that are produced by photoelectric effect, Compton scattering, and Auger effect is considered to be deposited in the electron emission position.

#### 2.1.3. gCTD (U Texas)

The gCTD package [10] is a GPU-based software that was coded under the Compute Unified Device Architecture (CUDA) platform that was developed by NVIDIA. The trajectory is calculated using the Woodcock tracking algorithm [35] that allows the simulation of the photon transport without the cumbersome ray tracing process and voxel boundary crossing checking. Since the range of those electrons is usually less than the voxel size in the keV energy range, the electron transport is not simulated. The GPU card that was used for the simulations was the NVIDIA GeForce RTX^TM^ 3090, a graphics card that was based on the NVIDIA Ampere architecture with 10,496 cores, boost core clock speed of 1.70 GHz, and 24 GB GDDR6X memory with 384-bit memory bus.

### 2.2. Case Studies

#### 2.2.1. Flat Field

**Figure 1:**
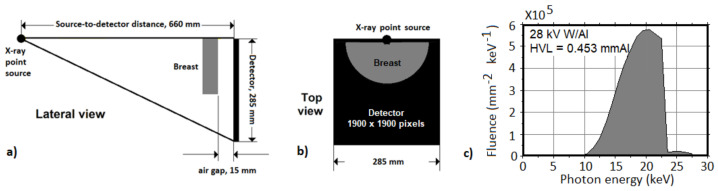
Scratch of the lateral view (**a**) and the top view (**b**) of the simulated setup for the flat field and uniform cylinder test. The X-ray source was at 595 mm (SOD) from the object and at 660 mm (SDD) from the 285 × 285 mm^2^ (1900 × 1900 pixel) detector. In (**c**) is shown the 28 kV W/Al X-ray spectrum used in these tests.

The first case study consisted of a simple flat field simulated irradiation without the object in the field of view. The X-ray source was simulated as an isotropic point source that was placed at 660 mm from the detector surface. The X-ray focal spot is aligned with the upper side of the detector (Figure 1a). In this geometry, the X-ray beam (28 kV W/Al, Figure 1c) is collimated at the detector surface to completely irradiate a 285 × 285 mm^2^ detector (1900 × 1900 pixel, pitch 0.15 mm). For each code the cumulative energy of the photons impinging on the pixels for 10^11^ launched photons was scored.

#### 2.2.2. Breast Object—Planar Imaging and Dosimetry

In the second case, we included a simulated test object in the simulated geometry. It was a a cylinder with semi-circular cross section with a radius of 100 mm mimicking 50-mm thick compressed breast, comprising 2 mm skin thickness. The inner portion of the breast was made of 20% homogeneous glandular tissue. The airgap (i.e., the distances between the detector surface and the lower surface of the breast) was set to 15 mm. We scored the cumulative energy per pixel (imaging test) and the dose deposition with a dose map resolution of 1 × 1 × 1 mm^3^ (dose test). For the simulations, we launched 10^11^ photons to have a statistical uncertainty of less than 1%. 

#### 2.2.3. Uniform Cylinder—CT Imaging and Dosimetry

**Figure 2:**
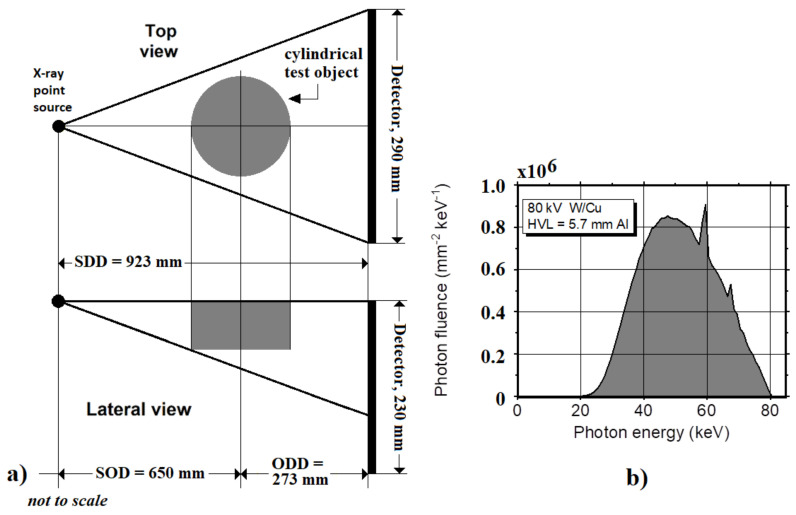
(**a**) The top and lateral views of the simulated CT setup. The X-ray source was at 273 mm (ODD) from the axis of rotation and at 650 mm (SOD) from the 290 × 230 mm^2^ flat panel detector. (**b**) The 80 kV W/Cu (HVL 5.74 mm Al) X-ray spectrum was obtained using TASMIP code [36] and used in this test.

To evaluate the dose distribution in a 3D imaging geometry, we simulated a setup that was based on the typical acquisition geometry that was used in all the research and commercial breast dedicated CT scanners where the patient is in a prone position, the breast hangs in a hole, and both the source and the detector turn around it [36]. For this work, the X-ray source was simulated as an isotropic source at 650 mm from the isocenter and the source–detector distance was 923 mm. The X-ray focal spot was aligned with the upper side of the detector. In this geometry the beam has a half-cone beam shape, to completely irradiate a 290 mm × 230 mm detector. Figure 2a shows the simulated geometry. The simulated X-ray spectrum, shown in Figure 2b (80 kV, W anode/Cu added filtration, HVL = 5.74 mm Al) was that adopted in the Albion BCT scanner in the first prototype at UC Davis [37] and was computed as suggested in ref. [36]. We simulated 360 projections (1° angular step over 360°) launching as many as 10^9^ photons per projection, for a total of 3.6 × 10^11^ photons for a complete scan with a corresponding to breast MGD of about 0.018 mGy for each virtual exam.

The breast was mimicked as a homogeneous cylinder with a diameter of 140-mm and height of 150-mm. The homogeneous material was a mix of 50% glandular and 50% adipose tissues (density 0.9819 g/cm^3^, fraction mass: 0.107 [H], 0.401 [C], 0.025 [N], 0.464 [O], 0.003 [P]). The voxel dimensions were 1 × 1 × 1 mm^3^. As a global assessment index for dose map comparisons, we adopted the 3D gamma index [38]. This tool combines two criteria including the percentage dose-difference and the distance to agreement (DTA) in millimeters. A gamma index ≤ 1 represents fulfilment of the criteria.

#### 2.2.4. Virtual Clinical Phantom—Dosimetry

**Figure 3:**
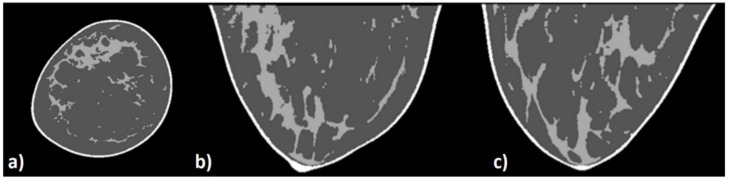
An example of an axial (**a**), sagittal (**b**), and coronal (**c**) slice of the patient breast after the voxel classification process.

Digital breast phantoms (Figure 3) [2] were derived from 3D clinical breast images acquired with the first BCT scanner at UC Davis (USA) [1,2]. Coronal slices are matrices of 512 × 512 pixel with pixel pitch ranging between 0.1938 mm and 0.4274 mm. The slice thickness ranges between 0.1907 mm and 0.2375 mm. Each of the image voxels was classified based on its content as air, glandular tissue, adipose tissue, or skin tissue. The composition of the simulated breast tissues (glandular, adipose, and skin tissues) was reported in [39]. The segmentation algorithm [22] classifies the skin voxel in each of the coronal slices by means of a local adaptive threshold algorithm. The glandular tissue was classified in each of the coronal slices by means of a threshold-based algorithm that was coupled with processes of erosion and hole-filling. Finally, the voxels within the skin border that not classified as glandular tissue were classified as containing adipose tissue. To guarantee the seamless structure of the glandular trees, processes of erosion and hole-filling were implemented in the direction that was perpendicular to the coronal planes. Figure 3 shows an example of an axial (Figure 3a), sagittal (Figure 3b), and coronal (Figure 3c) slice of the patient breasts of the adopted cohort after the voxel classification process. While the voxel content in the original image is expressed in Hounsfield Units (HU); the voxel of the produced digital breast phantom (Figure 3) can contain one out of four values representing one of the four main classified components. 

We simulated 360 projections that were uniformly spaced over a 360° angular span, with a total of 3.6 × 10^11^ photons for a complete scan (10^9^ photons launched per projection). For the radiation dose tests, we scored the dose that was deposited in the tissue components (skin, fat, gland) in 3D dose maps with a resolution of 1 × 1 × 1 mm^3^.

## 3. Results

### 3.1. Flat Field

**Figure 4:**
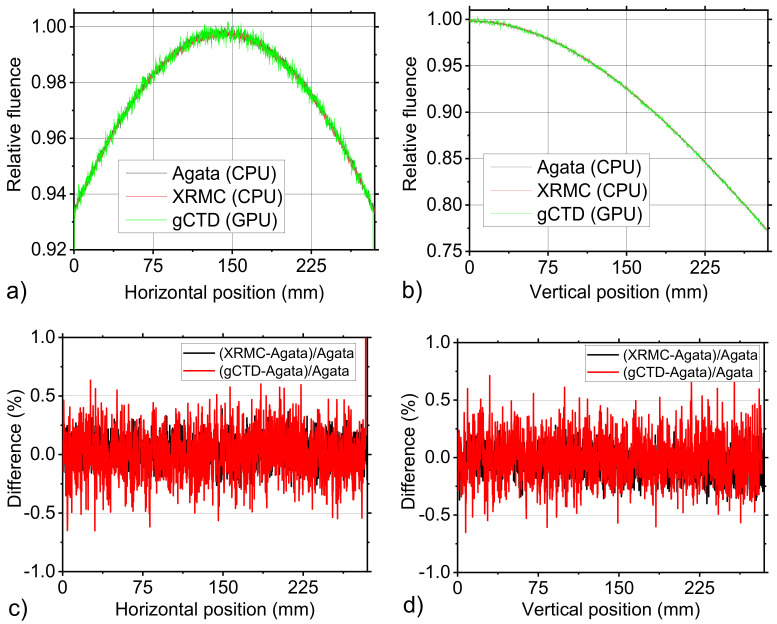
(**a**) Horizontal line profiles and (**b**) vertical line profiles of the normalized fluence values that were simulated with Agata, XRMC, and gCTD codes. The value was normalized to the highest value in the image. (**c**) and (**d**) are shown as the percentage difference between the values that were provided by the three simulation codes that were reported in (**a**) and in (**b**), respectively.

Using the irradiation geometry described in Section 2.2.1, a flat field irradiation was simulated by means of the three simulation codes. For each simulation the fluence value computed for each pixel of the detector was normalized with respect to the maximum value. Figure 4a,b report the horizontal and vertical line profiles that were measured in the same position of the simulated normalized fluence. Figure 4c,d show the difference, calculated pixel by pixel as indicated in the figure legend, between the measured values. Both in the horizontal and vertical directions the maximum difference value was about 0.5%.

### 3.2. Breast Object

**Figure 5:**
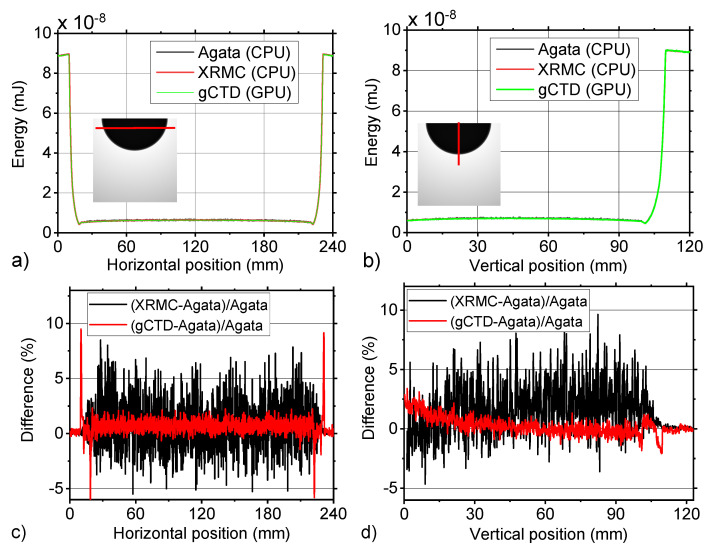
(**a**) Horizontal line profiles and (**b**) vertical line profiles of simulated deposited energy in each detector pixel with Agata Geant4, XRMC, and gCTD codes, respectively. The red lines indicate the position of the measured line profiles on the phantom images. (**c**) and (**d**) are shown as the percentage difference between the values that were provided by the three simulation codes that were reported in (**a**) and in (**b**), respectively.

Figure 5a,b report the horizontal and vertical line profiles on the simulated images of breast phantom. The simulated setup is the same that was used for the flat field case and the simulated breast phantom is described in Section 2.2. In each pixel of the image, we registered the cumulative impinging energy. The profiles were very similar as indicated also in Figure 5c,d which report the percentual difference between the profiles, calculated pixel by pixel. The maximum value of this difference (about 8%) was obtained when we compared the XRMC results with the Agata and gCTD data. Instead, the difference between the Agata and gCTD was reduced to only about 1%. In Figure 5c,d, we observed a systematic difference between the curves, although the difference levels were within an acceptable range for the cases we studied. We do not think it is due only to statistical fluctuation. Although fluctuations exist, the mean difference always lies above or below the x axis. It means that one software always records more energy in the detector than the other. This difference may be attributed to physics data such as cross section data, the way of interpolating phantom data. Another aspect is that the difference values vary with the vertical positions. For example, there exists a larger difference for gCTD at the edge part compared to Agata than that in the middle part. This may be attributed to different scattering simulation, which again is related to physics data.

**Figure 6:**
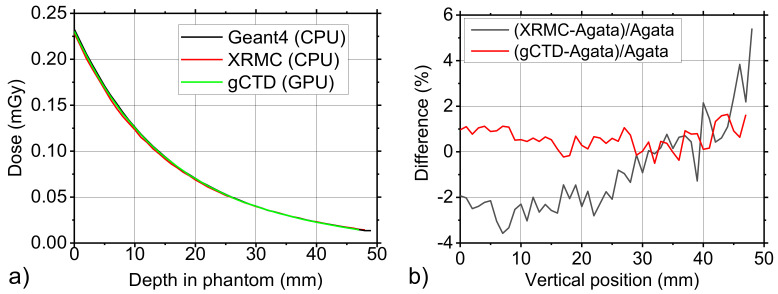
Line profiles of the simulated dose from the entrance surface to the rear of the breast phantom that was measured at the centre of phantom along the beam direction (**a**) and the percentage difference between the values that were provided by the different simulation code (**b**).

Figure 6a reports the line profiles of the simulated dose from the entrance surface to the rear of the breast phantom that was measured at the centre of the phantom along the beam direction. The profiles are quite similar, only a discrepancy is visible near the nipple position. This discrepancy is limited to a percentual difference of only 2% as reported in Figure 6b. It is also possible to note that in this case, there was a difference of only 1% between the Agata Geant4 and gCTD simulated data.

### 3.3. Uniform Cylinder—CT Dose

**Figure 7:**
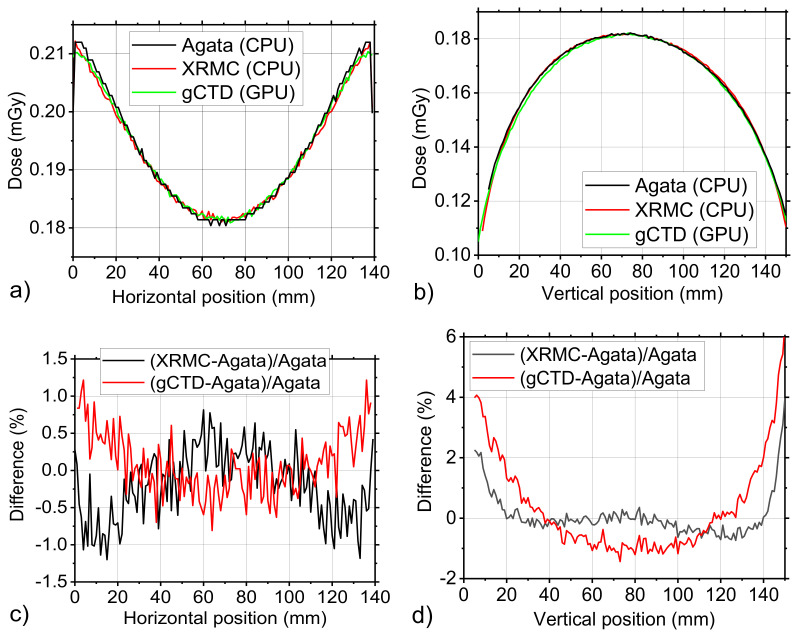
(**a**) Horizontal line profiles and (**b**) vertical line profiles of phantom dose distribution that was simulated with Geant4, XRMC, and gCTD code. (**c**) and (**d**) are shown the percentage difference between the values that were provided by the three simulation codes as reported in (**a**) and in (**b**), respectively.

To make a comparison in terms of 3D dose distribution, a complete CT irradiation of a uniform cylinder was simulated. The details of the simulated setup are reported in Section 2.2.3. The horizontal and vertical profiles evaluated in the central slice are reported in Figure 7a,b. In the horizontal direction, as indicated in Figure 7c, there is a very good agreement between the three codes (difference is less than 1%). In the vertical direction (Figure 7d), instead a 4–5% difference at the edges is shown. This difference is only 2–3% in the case of comparison between the Geant4 and gCTD code and only 1% between Geant4 and XRMC.

**Figure 8:**
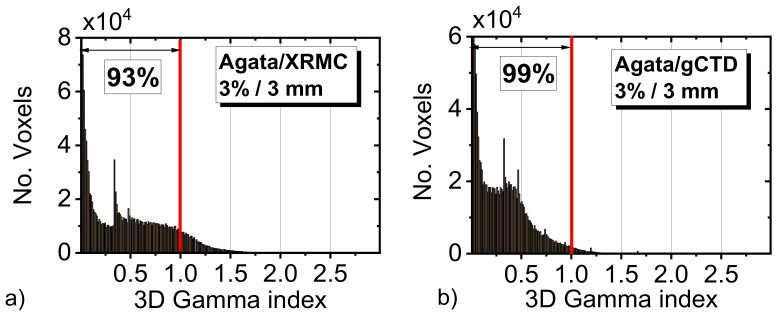
Distributions of the measured gamma indices in the comparison with the dose distribution that was simulated with the three different codes and different values for DTA and dose difference: (**a**) Agata (CPU) vs. XRMC (CPU), and (**b**) Agata (CPU) vs. gCTD (GPU). The vertical red line indicates the threshold value of 1 (93% and 99% for the two plots, respectively). Criteria considered for the 3D gamma index: 3%/3 mm.

For a more accurate evaluation of the agreement between the pairs of 3D dose maps that were obtained with different codes, we evaluated the 3D gamma index for each voxel. The gamma index was evaluated in the central 100 slices (100 mm phantom height), setting a value of 3 mm for distance to agreement criterion, and 3% for dose difference criterion. Figure 8 shows the whole histogram of these values. From the figure, it is possible to note that gCTD and XRMS simulations are in a good agreement with the simulated dose with Geant4 code. Specifically, we note that the 93% of the voxels for the XRMC simulation have a gamma value less than 1 (Figure 8a). For gCTD, instead, the number of voxels with a gamma factor that is less than 1 is about 99% (Figure 8b), indicating a slightly better agreement with the GPU-based code.

### 3.4. Anthropomorphic 3D Digital Breast Phantom

A simulated exam with a anthropomorphic breast phantom was realized with the gCTD code. The patient-derived anthropomorphic digital breast phantom that was used in this test was derived from a clinical breast CT scan at 80 kV, as reported in Ref. [2]. The phantom volume was 816.8 × 10^3^ mm^3^ (385 × 385 × 214 pixel with voxel volume 0.3539 × 0.3539 × 0.2056 mm^3^) and the phantom glandular fraction was 17% by weight.

**Figure 9:**
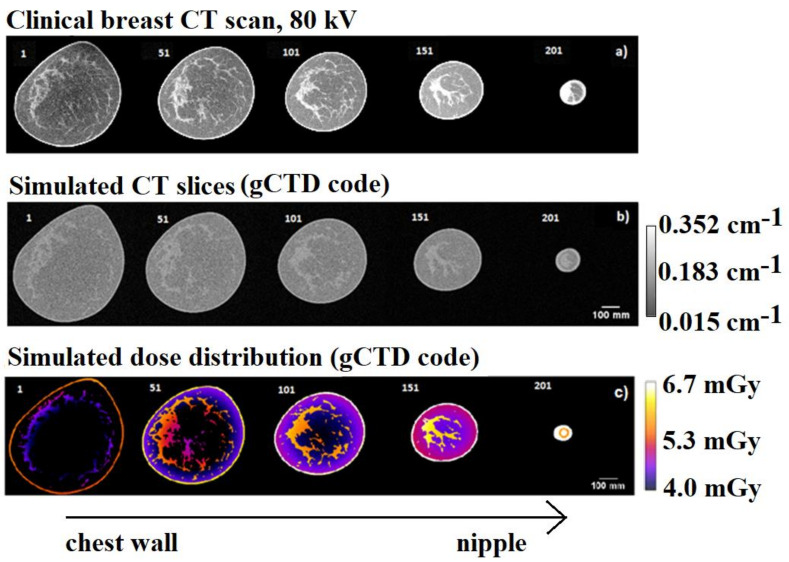
Example axial slices (#1 to #201) of (**a**) clinical breast CT scan at 80 kV and (**b**) simulated CT-reconstructed slices (FDK algorithm) that were obtained using the gCTD simulation code. In (**c**) are the reported calculated dose distributions in the corresponding slices.

The first raw images of Figure 9 show five different slices of the clinical breast image at different distances from the chest-wall (as reported in Figure 9a). The middle row (Figure 9b) shows the equivalent simulated reconstructed slices with the ASTRA toolbox using a FDK algorithm [40]. For the reconstruction we used the simulated projections by gCTD code starting from the segmented breast phantom. The lower raw (Figure 9b) shows the dose distribution of the same slices. The mean glandular dose was evaluated as 5.04 mGy (with a standard deviation of 0.6 mGy of the glandular dose distribution over the entire breast volume). These simulations were completed in about 360 s (GPU time). We estimated that the same simulation with the Agata Geant4 and XRMC code would take about 120 days or 4 days of computing time, respectively.

## 4. Discussion

Clinical trials are fundamental steps in the design, validation, and implementation of new medical imaging technology. These trials are often not practical due to ethical limitations, expense, time requirements, and difficulty in recruiting enough subjects. A possible alternative are VCTs. Several MC codes have been developed for breast imaging studies, for simulating images, and estimating organ doses [4,41,42,43,44,45,46,47,48,49]. Some representative examples are the Cancer Research UK-founded OPTIMAN project (https://medphys.royalsurrey.nhs.uk/nccpm/?s=optimam-sub (accessed on 10 February 2022)) to evaluate the smallest detectable diameter of various lesions in mammography and tomosynthesis [18,50] or the VICTRE project to compare the performance of DBT and DM in the detection of calcifications and masses under clinically realistic conditions [51].

A fundamental aspect for the success of the VCTs is related to the possibility to have fast simulation process. Monte Carlo-based codes, although accurate, are computationally too slow particularly for applications such as tomosynthesis or breast CT. To improve this aspect, ray-tracing algorithms have been developed [4,52] in which only the analytical approximation of X-ray-tissues is estimated using the Beer–Lambert law. Other possible techniques have been proposed to speed up these trials such as the variance reduction technique or the use of GPUs [9,10,53,54].

In this work, we compared an MC-based code (Agata code) and a simulation code implementing a variance reduction technique (XRMC code) and a simulation code that was developed on a CUDA platform (gCTD). The proposed case studies showed good agreement (about 3–4%) between the codes but with a remarkable computational time reduction (Table 1). This little difference is mainly due to the differences among the software of physics cross section data, the way of interpolating phantom data, and the physics data. For example, in the case of a uniform cylinder CT case study, we have a computational time of about 2300 s, 1100 s, and 1 s (per 10^9^ photons launched) for the Geant4 code, XRMC code, and gCTD code, respectively. We expect a computation time of about 550s running the code XRMC on the same hardware that was used for Agata. Besides advantages in speed, GPUs also allow for the reduction of cost and can be locally hosted and managed. In fact, for the Geant4 code we used a cluster of four servers while per gCTD we used a single NVIDIA GeForce RTX^TM^ 3090 graphics card that was hosted in a desktop PC. In the last case study, it showed the possibility to realize a complete imaging and dosimetric virtual study. In the case of a BCT virtual scan, a GPU computing time of 10^4^ s (2.8 h) will be needed for 10^13^ photons that are launched in the simulation, generating 360 views (1900 × 1900 pixel, pitch 0.15 mm) and 3D dose maps (ca. 5 mGy MGD) with a voxelized breast phantom (ca. 32 mega voxels of total volume ca. 93 × 93 × 93 mm^3^). When considering that the GPU-based farms are readily available at relatively low costs, this performance is promising also in view of real-time on-site VCTs applications for DM, DBT, and BCT studies. In these studies, the use of a GPU-based farm would allow, for example, the real-time evaluation of the dose that is delivered to the patient during the exam. In fact, as showed in Section 3.4, we could use the 3D image of the patient that is acquired in the CT exam to evaluate the dose that is delivered to the patient for acquiring the image itself.

## 5. Conclusions

We demonstrated the realization of accurate and fast virtual studies in X-ray breast imaging using CPU-based as well as GPU-based MC simulation codes. We realized a direct comparison of test cases between the three simulation codes that were based on Geant4 (Agata) without variance reduction, a code (XRMC) implementing a variance reduction technique, and a code (gCTD) implementing CUDA programming on a performance, low-cost GPU card. The Agata code that was already used in previous studies was used as a benchmark, with discrepancy of the numerical results for the other two codes being in a range of 3–4%. In the case of a BCT virtual exam, a complete imaging and dosimetric virtual study can be obtained in about 2.8 h on a single high-performance GPU. This virtual exam would replicate the image noise and MGD of a real BCT clinical scan.

## Figures and Tables

**Table 1:** Summary of the used software and hardware and computation times that are estimated for the case studies in sect. 3.3. The phantom volume was 8.16 × 10^5^ mm^3^.

Software Code	Computer Hardware	Computing Time (s/10^9^ photons)	Computing Time (s/10^9^ photons/mm^3^)
Agata (Geant4 v10.6 patch 01) (CPU-based)	2 x AMD EPYC 7281, 2.2 GHz, 32-Core Processors, 256 GB RAM	2300	2.816 × 10^−3^
XRMC (CPU-based)	Intel Core i9-9700K 8-Core Processors, 3.6 GHz	1100	1.347 × 10^−3^
gCTD (GPU-based)	NVIDIA GeForce RTX^TM^ 3090	1	1.224 × 10^−6^

## Data Availability

Software Availability: The Agata, gCTD, and XRMC codes are open source and have not been published online. All users could use them freely as they are upon reasonable request to Antonio Sarno, Xun Jia and Bruno Golosio, respectively.
